# Catalytic Oxidation of CO and Benzene over Metal Nanoparticles Loaded on Hierarchical MFI Zeolite

**DOI:** 10.3390/molecules26195893

**Published:** 2021-09-28

**Authors:** Totka Todorova, Petya Petrova, Yuri Kalvachev

**Affiliations:** Institute of Catalysis, Bulgarian Academy of Sciences, Acad. G. Bonchev St., bl. 11, 1113 Sofia, Bulgaria; petia@ic.bas.bg

**Keywords:** ZSM-5 zeolite, noble metals, hierarchical materials, catalysts, CO oxidation, benzene oxidation

## Abstract

In order to obtain highly active catalytic materials for oxidation of carbon monoxide and volatile organic compounds (VOCs), monometallic platinum, copper, and palladium catalysts were prepared by using of two types of ZSM-5 zeolite as supports—parent ZSM-5 and the same one treated by HF and NH_4_F buffer solution. The catalyst samples, obtained by loading of platinum, palladium, and copper on ZSM-5 zeolite treated using HF and NH_4_F buffer solution, were more active in the reaction of CO and benzene oxidation compared with catalyst samples containing untreated zeolite. The presence of secondary mesoporosity played a positive role in increasing the catalytic activity due to improved reactant diffusion. The only exception was the copper catalysts in the reaction of CO oxidation, in which case the catalyst, based on untreated ZSM-5 zeolite, was more active. In this specific case, the key role is played by the oxidative state of copper species loaded on the ZSM-5 zeolites.

## 1. Introduction

The main atmospheric pollutants that have a direct impact on human health are Volatile Organic Compounds (VOCs) and CO. The VOCs are a large group of hydrocarbon compounds that are easily released into the air at atmospheric pressure and room temperature. Waste gases can contain a variety of volatile hydrocarbons, alkanes, alcohols, ketones, aldehydes, aromatics, organic acids, ethers, aldehydes, and others. Their emissions into the environment originate mainly from the petroleum and chemical industry, the production of solvents, cleaning products, printing machines, and others [1,2,3]. In particular, benzene is one of the most dangerous and carcinogenic representatives of VOCs due to its aromatic nature and high structural stability, requiring extreme experimental conditions for its removal [4,5]. Another type of toxic gas, odorless, colorless, and tasteless, that is especially important to emission control is CO—it is also called the silent killer. It is well known that even very small amounts of CO in the air can be fatal, since the carbon monoxide molecules have an affinity for binding to hemoglobin in blood cells, replacing oxygen. In addition to waste gas treatment and automotive emissions, the CO oxidation reaction is often used as a test reaction to monitor the efficiency of a catalyst. Therefore, it is a crucial issue to minimize VOCs and CO emissions in the environment [6,7]. Among the studied methods and techniques, catalytic oxidation is the most popular due to its operating flexibility for a number of organic compounds under soft operating conditions in combination with low energy use and destructive efficiency [8,9]. Although a significant number of industrial catalysts have been developed for the oxidation of CO and VOCs, the creation of some new ones and optimization of the already existing ones is an important goal of scientists due to the great variety of organic molecules and complex nature of organic mixtures found in practice. The successful commercial catalysts can be classified into three groups: (1) noble metal catalysts; (2) oxides of transition metal catalysts; and (3) mixed-metal catalysts [10]. The characteristics of catalysts, based on noble metals, are their specific activity, resistance to disinfection, and ability to regenerate. The catalysts, containing platinum (Pt) or palladium (Pd), are the most commonly used ones to promote catalytic oxidation reactions. Despite their good efficiency, serious problems are posed by their high cost, limited availability, and sensitivity to high temperatures and poisons, which necessitate their replacement by catalysts containing transition metals such as: manganese (Mn), nickel (Ni), chromium (Cr), copper (Cu), cobalt (Co), and others. The activity of the loaded metal catalysts strongly depends on the method of preparation, the type of precursors, the method of metal loading, the particle size, and the nature of the carriers [11,12,13].

It was found out that applying noble metals and metal oxides on support materials having a large active surface area and specific physicochemical properties results in the reduction of the amount and the increase in degree of dispersion of metal phase into the catalyst without changing its activity. Al_2_O_3_, SiO_2_, ZrO_2_, TiO_2_, zeolites, and others have been often used as support materials [14,15,16]. The zeolite family is a suitable candidate for catalysts in the oxidation reaction of hydrocarbons due to their characteristic crystalline microporous structure, having a high surface area to pore volume ratio, humidity resistance, and thermal and acid stability [17,18]. Zeolite ZSM-5 is a well-known high-silicon crystalline aluminosilicate with wide application both as a catalyst and as a sorbent. Its chemical formula is |Na^+^_n_(H_2_O)_16_|[Al_n_Si_96-n_O_192_], where n < 27, and its structure is composed by five membered rings, forming the composite building units “mfi”, “mor”, “cas”, and “mel”. By combining the building units, a three-dimensional structure is formed, containing mutually intersecting 10-membered channel systems [19,20,21]. Although it belongs to the group of medium porous zeolites, ZSM-5 has a relatively high resistance to coke formation [22,23,24]. Pt and Pd impregnated ZSM-5 catalysts are the most effective for purification of waste gases of chlorine-containing organic molecules, double bonded compounds, aldehydes, and etc., which are considered to be among the most disturbing indoor pollutants even in very small amounts [25,26,27]. The characteristics of these catalysts are their ability to adsorb toxic molecules on the active crystal surface, to store chemical intermediates, and their high selectivity for the formation of CO_2_ and H_2_O as end products instead of CO. There is a known case in which the ZSM-5 surface was previously F-impregnated with subsequent deposition of metal phase for improving of water tolerance of the catalyst, and its stability and lifetime during the processes [25]. The oxidative activity of Pt/ZSM-5 systems is efficient enough and it is even used to convert diesel soot into safe components for nature and humans [28]. The ability of the Pt/H-ZSM-5 catalyst to oxidize soot was studied in both O_2_ and NO + O_2_ gas mixtures. Two main factors influence the oxidation reaction. First was the ability of the acidic zeolite support to promote the adsorption of NO_2_ on the catalyst, which helps to preferentially attach the soot to NO_2_ molecules, providing better opportunities for NO_2_-soot reactions. The second factor was the strongly acidic sites, formed by Pt metal nanoparticles, which participate in the catalytic reaction by formation and decomposition of surface oxygenated complexes. In order to obtain zeolite catalysts having a large active surface area and the presence of more pores, Chi He et al. impregnated Pd nanoparticles onto composite materials containing two types of zeolites (ZSM-5 and MCM-48) with different acidities [29]. Thus, the so-formed catalysts showed good activity in the complete elimination of benzene due to Pd^0^ and Pd^2+^ species, which were formed predominantly on the acidic supports.

In order to reduce the final cost of catalytic materials, Xin Xing et al. monitored the effect of various Cu-containing supports (ZSM-5, MOR, MCM-22, Hβ, and SAPO-34) in the reaction of selective catalytic oxidation of n-butylamine [30]. In their study, they reported that the different zeolitic structures significantly influenced the existing forms and dispersion of Cu species, changing the redox properties of the catalysts, and among them all Cu/ZSM-5 showed the best behavior in the reaction under consideration. A series of studies have focused on Cu active sites, their amount, acidity, distribution and condition (Cu^2+^, Cu^+^ and/or Cu^0^), and their effect on activity in catalytic oxidation reactions of n -butylamine, acrylonitrile decomposition, and Water-Gas Shift reaction [31,32,33,34]. The results show that the formation of Cu^2+^ is strongly influenced by the Si/Al ratio of the zeolite structure and the amount of the metal phase. Upon increasing SiO_2_/Al_2_O_3_ ratio above 25 the selectivity to N_2_ decreases, which is closely related to the Cu^2+^ species. Moreover, upon increasing the Si/Al ratio, the isolated Cu^2+^ forms decreased, and the oligomeric Cu^2+^-O^2−^-Cu^2+^ species are being formed predominantly. However, in reactions of NO decomposition on the Cu/ZSM-5 catalyst, reversible poisoning of the catalyst by NO and oxygen and its rapid deactivation has been observed [35].

From all these facts, it becomes clear that for the production of catalysts possessing the desired properties and activity, many parameters must be taken into account, such as: type of carrier material (crystal structure, active surface area, presence or absence of pores), metal phase, dispersion of metals, type of organic compounds, and experimental conditions. However, looking at a given chemical reaction on the molecular level, it becomes clear that the diffusion properties of the supports are essential parameters, that is, the transport of reagents and products to and from the active catalytic centers. Some of these diffusion limitations could be avoided by choosing zeolite materials as carriers in the formation of catalysts having wide applications. Their open structure allows interaction with the surrounding environment, both with the external and internal surface area. Although ZSM-5 has a three-dimensional, open structure with comparative resistance to coke formation, it is known that during the temperature conversion of hydrocarbons, the catalysts decrease their activity due to coke clogging and lack of access of reagents to the active sites. The passage of reagents and products through the catalyst can be significantly improved by creating of additional micro- and/or meso-pores on the support surface. The creation of hierarchical zeolites can be carried out by two methods: the first one is during zeolite synthesis (in-situ), and the second one is through post-synthesis methods and techniques (post-situ). There are only a few articles related to the creation of ZSM-5 supports containing additional porosity and its influence on catalytic combustion reactions of VOCs and CO. Using an in-situ technique for the production of hierarchical ZSM-5 zeolite as a carrier, Fujian Liu et al. reported the formation of effective and long-lifetime bi-metallic catalysts for benzene combustion [36]. During the synthesis process, they added a copolymer to the reaction mixture and, by high-temperature treatment of the crystalline product, the organic molecule was burned out, yielding a mesoporous zeolite structure. Serious disadvantages here include the high cost of organic structure-directing agents, the release of emissions, and the time and energy required for their combustion. The alternatives of these attempts are post-synthesis methods and techniques. Among post-synthesis methods, the most commonly used ones are chemical treatment with various bases or acids and steam treatment. Sibei Zou et al. applied etching of ZSM-5 with nitric acid to prepare Pt mesoporous catalysts tested in toluene combustion [37]. The molecular size of toluene was about 0.67 nm, which is very close to the pore size of parent zeolite, and it complicates the reactions. The introduction of additional porosity leads to a significant facilitation of the diffusion properties and an increase of the catalyst efficiency in the selected reaction. During the treatment with steam and acidic solutions the extraction of aluminum atoms out of the zeolite structure (dealumination) [38,39] takes place, while when treated with alkaline solutions, removal of silicon tetrahedra is performed, preferentially (desilication) [40,41]. These approaches showed selectivity to one of the both skeletal elements, that is, the Si/Al ratio of the parent zeolite structure changing Brönsted and Lewis acidity, which may affect the activity of the final catalysts [42,43]. The chemical treatment by fluoride ions is an interesting approach for creating secondary porosity in aluminosilicate zeolite structure [44]. This method was used in the present work and the choice was based on the statement that the formed HF_2_^−^ anion has a high reactivity and it does not show any selectivity to either of the skeletal elements (Si or Al). It is known that in aqueous solution of HF containing different types of fluorides, to ensure a chemical equilibrium shift to obtain more active HF_2_^−^ anions, F^−^ ions must be introduced into the system via NH_4_F buffer [45]. The zeolite dissolution by HF_2_^−^ initially affects the structural defects and/or the adhesion positions of the individual crystallites and it continues deep inside the crystals. The obtained hierarchical samples are expected to be a suitable support material for the deposition of active metals to form catalysts in reactions of complete oxidation of volatile organic compounds (VOCs). The obtained material will possess a number of advantages: high surface area, reduced coke formation (slower deactivation of the catalyst), facilitated movement of initial and final products, and its prevention extraction of the active metals from the catalyst structure [46,47,48]. The fluorine etching was tested onto a series of ferrierite crystals and mordenite type of zeolites. In our previous work, we investigated in detail the catalytic activity of mordenite-type of zeolite in the reaction of m-xylene transformation after acidic treatment with HF in combination with NH_4_F. It was proved that the treated samples have chemical characteristics very close to their parent counterparts, but some differences were observed in the catalytic activity, where the hierarchical samples demonstrated significantly higher conversion [49,50]. By impregnation of Zr over hierarchical mordenite crystals, we obtained catalysts with higher catalytic activity in glycerol esterification with acetic acid and selectivity to valuable triacylglycerol [51]. The structural and morphological changes of Al and Ga analogues of ZSM-5 as a result of HF_2_^−^ treatment were also monitored in reaction of m-xylene transformation [52].

The present work investigated all the stages of catalysts formation, acquiring a large active surface area and stable catalytic behavior in reactions of complete oxidation of benzene and CO. In order to obtain an effective zeolite support, ZSM-5 zeolite was successfully synthesized by hydrothermal synthesis, followed by treatment with HF acid in combination with NH_4_F. The features of these supports are combination of the properties of the parent samples: crystalline structure, chemical composition, thermal and acid resistance, and the specifications of the hierarchical materials: larger pore size, overcoming the transport problems of reactants and products, easy access to the internal surface area of zeolite, large active surface area, and etc. By a wet impregnation method, a series of metals, Pt, Pd, and Cu, as active phase were successfully dispersed. Comparative analyses were performed between parent and hierarchical catalysts. Series of physicochemical methods such as XRD, physical adsorption/desorption of N_2_, TEM microscopy, TPR, and gas chromatography were used to characterize the resulting catalysts.

## 2. Results and Discussion

**Figure 1:**
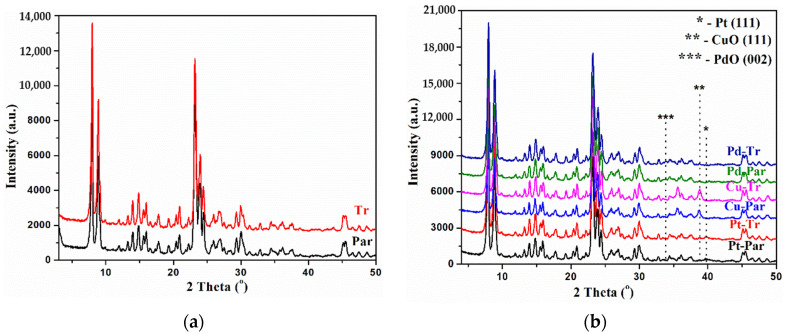
XRD patterns of (**a**) parent and treated ZSM-5 samples; (**b**) parent and treated impregnated with Pt, Cu, and Pd.

Figure 1a represents the X-ray diffraction patterns of the hydrothermally synthesized zeolite ZSM-5 (Par) and the one treated with an aqueous solution of 0.25 M HF and NH_4_F zeolite ZSM-5 (Tr). The X-ray pattern for the parent sample contains all signals corresponding to the ZSM-5 phase with a high degree of crystallinity. Despite the acidic treatment, the crystallinity and structural characteristics were preserved in hierarchical samples. Pt, Cu, and Pd were deposited on parent and on the treated supports by the wet impregnation method and the X-ray patterns are shown in Figure 1b. The three series of secondary porosity catalysts (Pt, Cu, and Pd) had diffractograms very similar to their parent counterparts. Both Pt-containing samples, in addition to the zeolite phase, contained a very weak signal, about 2θ ≈ 39.5, corresponding to platinum metal, while in the Cu samples a signal of about 2θ ≈ 38.6, corresponding to the presence of CuO phase, was observed. In the diffractograms of the Pd and Pt-deposited samples, there were no additional signals corresponding to oxidized forms (for PdO 2θ ≈ 33.8; for PtO and/or PtO_2_ 2θ ≈ 27.5 and 2θ ≈ 34.4), but this is not a characteristic for the absence of PdO and PtO_x_ nanoparticles due to the overlapping signal position of the metal oxides and zeolite phase [53,54,55,56]. The low intensity and/or absence of signals in the Pt and Pd X-ray diffraction patterns can be attributed to fine dispersion of metallic species on the zeolite supports (evidenced also by TEM).

**Figure 2:**
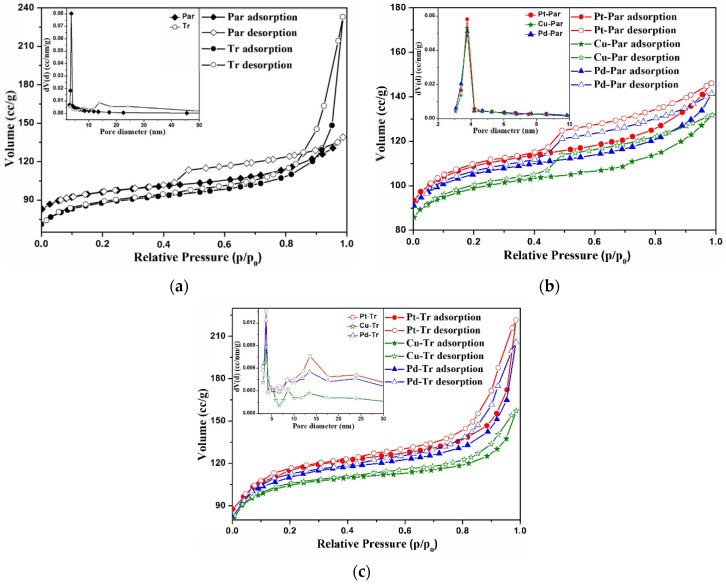
Nitrogen adsorption/desorption isotherms of (**a**) parent and treated; (**b**) parent impregnated with Pt, Cu, and Pd; and (**c**) treated impregnated with Pt, Cu, and Pd. Inset—corresponding pore size distribution.

The specific surface areas and micro-/mesopore volumes of parent, treated, and metal-loaded ZSM-5 were analyzed by a physical nitrogen adsorption (Table 1 and Figure 2). Typically, for microporous materials, all the samples formed isotherms of type I. The higher nitrogen uptake was observed in the isotherms of the samples after acid attack (Figure 2) at partial pressure close to 1 as a result of the secondary porosity and reduced crystallite size. The metal-loaded initial catalysts demonstrated relatively uniform nitrogen adsorption at high partial pressure values (Figure 2b), whereas the acid-treated samples with applied active metals showed significantly improved physical nitrogen absorption at the same pressure values (Figure 2c).

The results of nitrogen adsorption/desorption isotherms were used to obtain the specific surface areas and pore sizes of the initial and modified catalysts (Table 1). After the etching process, changes in the textural properties (specific surface area and pore volume) of the treated sample were observed. As a result of the applied acidic treatment, decreasing of the specific surface area and micropore volume was observed, while the volume of the mesopores increased significantly. Typical for the samples after metals deposition is an increase in the pore volume and the specific surface area. However, in the case of acid-treated catalysts this effect was reinforced, i.e., an additional increase in the active surface and the porous volume was observed, regardless of the amount and type of applied metals. It can be seen that the sample subjected to acidic treatment increased the volume of the secondary mesopores more than two-fold compared with the parent sample. After metals depositions over the parent and treated zeolite supports, a decrease in the volume of meso- and macropores was observed, especially in the case of Cu-catalysts due to the larger amount of the metal component. However, the treated catalysts retained a larger volume of meso- and macro-pores compared with the catalysts formed with the parent zeolite. This effect can be explained by blockage of the zeolite channels after the metals deposition on parent sample, while the hierarchical zeolite support provides a larger active surface area and additional micro- and/or meso-pores allowing even distribution of the metal clusters.

**Figure 3:**
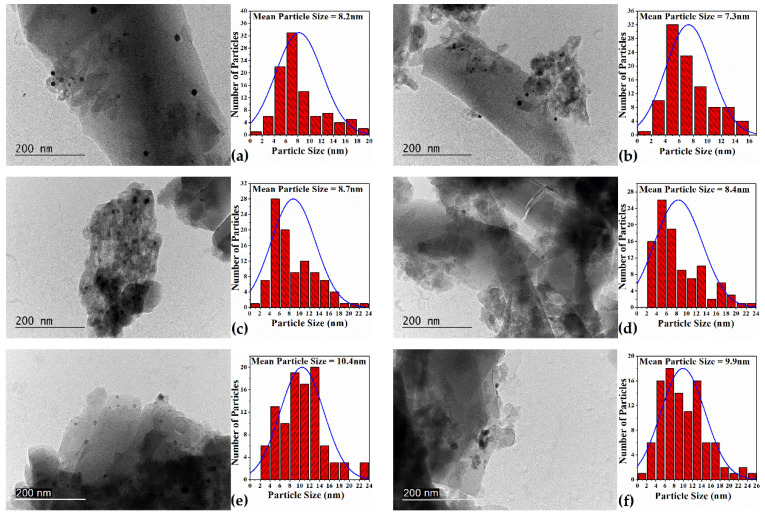
TEM images of parent and treated impregnated samples: (**a**) Pt parent, (**b**) Pt treated, (**c**) Cu parent, (**d**) Cu treated, (**e**) Pd parent, (**f**) Pd treated and its corresponding particles’ size distribution.

TEM images (Figure 3) provide information about zeolite morphology, size, and distribution of metal nanoparticles. The effect of the post-synthesis acidic treatment onto the zeolite structure is represented in the micrographs. In parent ZSM-5 samples, aggregates of zeolite crystals, on which the metal component was applied, were reported, but in hierarchical analogs significant destruction of both aggregates and individual crystals was observed with formation of crystals with reduced sizes. The reason for this is the high activity of HF_2_^−^, randomly attacking Si and Al from the zeolite structure and forming a secondary porosity, but it initially affected the structural defects and adhesion positions of the individual crystals, breaking them into smaller pieces. The newly formed crystals had a larger active surface area, formed by the combination of reduced crystal size and additional pores, improving the diffusion properties of the zeolite. All the catalysts were obtained by wet impregnation of the metals on parent and acid-treated ZSM-5 supports. By measuring the sizes of 100 randomly selected particles in the corresponding TEM images, the particle size distribution histograms (Figure 3a–f) were obtained [57]. The histograms revealed a close distribution of metal nanoparticles on the initial and treated counterparts. A difference was observed in the histograms at the average particle size, where on the acid-treated catalysts (Pt-tr, Cu-tr, and Pd-tr—Figure 3b,d,f) the metal nanoparticles showed lower values compared with the non-hierarchical ones (Pt-par, Cu-par, and Pd-par—Figure 3a,c,e). The largest difference was observed in platinum-containing catalysts, where the average size of the metal phase onto the parent zeolite was about 8.2 nm, but in the acid analogue this value was about 7.3 nm. However, for catalysts having the other two types of metals, this difference in the average size between parent and treated supports was only 0.5 nm. In addition to the lower values of the mean metal particle size, there was also an increase in the number of particles with sizes within the range of 2–10 nm at the expense of larger metal aggregates at the treated catalysts. This fact is in agreement with the statement for a finer distribution of metal particles on the hierarchical samples.

**Figure 4:**
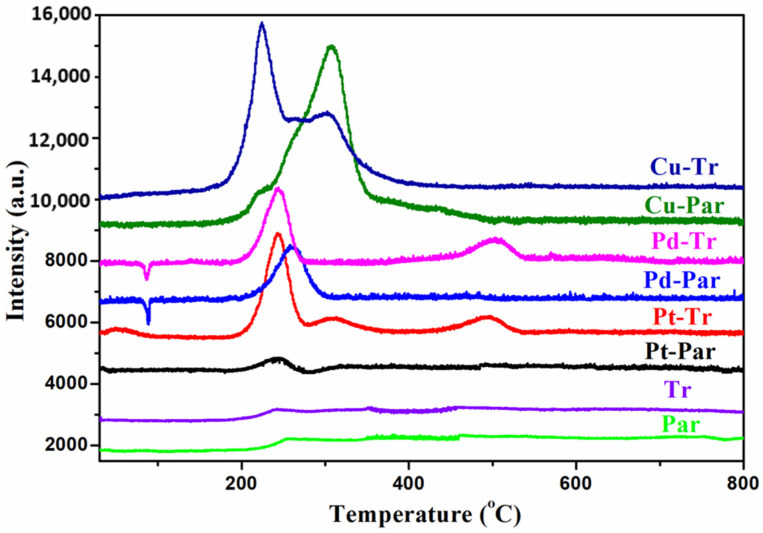
TPR profiles of studied samples: Pt parent, Pt treated, Cu parent, Cu treated, Pd parent, and Pd treated.

An indicator of the activity of the catalysts is their reducibility, investigated by the H_2_ temperature-programmed reduction method (TPR—Figure 4). In the H_2_-TPR profile of the Pt-parent catalyst, a low-intensity reduction peak, corresponding to the reduction of Pt^2+^ ions to Pt^0^ on the catalyst surface, was observed. On the other hand, the low-intensity can be attributed to the metallic Pt phase (Pt^0^), which was formed during the high-temperature catalyst preparation process (calcination) along with a small amount of the Pt^2+^ species. However, for the Pt-treated catalyst, three reduction signals were observed. The low temperature peak 190–280 °C can be attributed to reduction of the surface-located Pt^2+^ forms, the second peak 280–350 °C corresponds to the reduction of Pt species interacting with support surface, and the highest 450–530 °C to the reduction of Pt species strongly interacting with the zeolite support [58,59,60]. The different reduction behavior of Pts loaded over acid-treated ZSM-5 can be explained by the reduction of the crystallite sizes and formation of secondary porosity, leading to stronger Pt-support interactions. Regardless of the higher intensity of the low-temperature peak, it can be explained by the lower oxidative decomposition of Pt-salt to Pt^0^ species during the calcination process, forming more Pt^2+^ ions.

A characteristic feature of the TPR profiles of both Pd-zeolite catalysts is the presence of a negative peak within the temperature range 75–90 °C as well as a peak corresponding to H_2_ consumption within the range 190–300 °C. It is well known that PdCl_2_ and/or PdO are easily reduced at room temperature to Pd metal particles and they react with hydrogen to form PdH_x_ species. The low-temperature negative peaks were formed as a result of the decomposition of this PdH_x_ species releasing H_2_ during the initial stage of the experiment [29]. The increased intensity and position of the decomposition peak can be assigned to decreased of Pd dispersion over support zeolite. The second intensive peak corresponds to a high H_2_ consumption and it was assigned to the reduction process of Pd^2+^ (PdO) species into metallic Pd (Figure 4, Pd-par and Pd-tr). A difference between the parent and treated Pd sample was observed at a temperature of 450–540 °C, wherein the hierarchical analogue showed a signal associated with the reduction of Pd forms that interact strongly with the zeolite support, while the parent catalyst had no peak within this temperature range [36,61]. In the TPR curve of copper-loaded samples, two resolved reduction peaks within the temperature interval 170–380 °C were observed. The two peaks could be assigned to the stepwise reduction of CuO (CuO → Cu_2_O → Cu). According to previous reports [62], the mechanism of copper-impregnated catalysts proceeds through the following steps:

(1) CuO + H_2_ → Cu^0^ + H_2_O
(2) Cu^2+^ + 0.5H_2_ → Cu^+^ + H^+^
(3) Cu^+^ + 0.5H_2_ → Cu^0^ + H^+^

The reactions (1) and (2) occur within a lower temperature range, while reaction (3) occurs at higher temperatures. In the TPR profile, curves of our two samples (parent and treated) containing copper as an active phase showed a low-temperature reduction peak corresponding to CuO reduction into Cu^0^ and Cu^+^ species at the same time. The presence of a second peak within the temperature range 240–380 °C was assigned to the reduction of Cu^+^ into Cu^0^. There was a difference between the two copper catalysts in the intensities of the peaks. In the parent sample, a higher consumption of hydrogen was observed at higher temperatures, while in the treated sample, the low temperature of the signal had higher intensity. This fact is in a good agreement with the redox properties of Cu cations, which are controlled by the local Si/Al ratio and structural morphology of the support zeolites, influencing their reducibility [63]. For all the treated catalysts (Pt-tr, Cu-tr, and Pd-tr), the reduction peaks were shifted towards lower temperature, which means they showed better redox ability and better activity at low temperatures compared with their untreated analogues.

**Figure 5:**
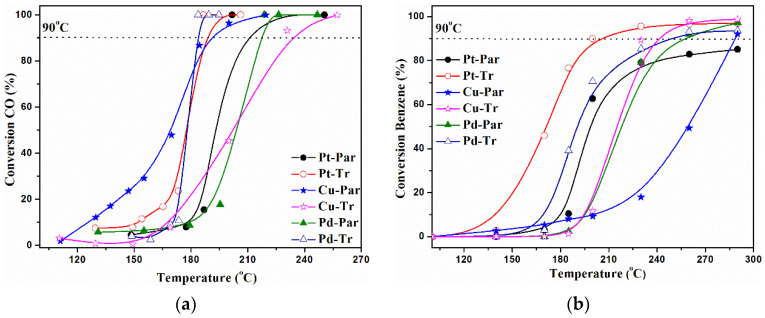
Temperature dependence of (**a**) carbon monoxide and (**b**) benzene conversion.

The temperature dependences of carbon monoxide and benzene oxidation over the investigated catalysts are shown in Figure 5. In the case of benzene oxidation, H_2_O and CO_2_ were the only detected reaction products in all studied samples. The samples were compared according to the temperature for 90% reagent conversion or according to that one for maximum of conversion in cases this value was not reached. The following order of activity in the reaction of CO oxidation was established as follows: Pd-Tr > Pt-Tr > Cu-Par > Pt-Par > Pd-Par > Cu-Tr (see Table 2). The row of activity in the complete benzene oxidation is the following: Pt-Tr > Cu-Tr > Pd-Tr > Pd-Par > Cu-Par > Pt-Par (85%, 290 °C). The value in brackets for the last sample shows the maximal conversion reached within the studied temperature interval 100–290 °C. The sample Pt-Par had the lowest reducibility (see Figure 4).

It is obvious that the preliminary treatment of the support has a crucial role on the catalytic activity of the investigated samplesthe catalysts with treated support were more active. The effect was more pronounced in the reaction of benzene oxidation. The size of benzene molecules was higher than the size of CO molecules and the presence of secondary mesoporosity had a bigger effect. The diffusion problems that could occur during the oxidation of benzene on a parent samples did not exist in the cases of treated samples. Benzene molecules improved access to the active catalytic sites.

In CO oxidation, also the catalysts based on the treated support were more active. The only exception were copper catalysts. CuPar, the catalyst with parent support, was more active for CO oxidation than CuTr. Copper can exist in different oxidation states such as Cu^2+^, Cu^1+^, and Cu^0^. These copper species can coexist together at different ratios depending on many factors such as preparation method and reduction process. These copper species have different CO oxidation activities. It is known that Cu^1+^ is very effective in CO oxidation compared with Cu^2+^ [64]. This is in good agreement with our TPR results, which showed preferable presence of Cu^1+^ in the sample CuPar rather than Cu^2+^. This is the main reason for the higher activity of the CuPar sample.

## 3. Materials and Methods

### 3.1. Catalysts Preparation

ZSM-5 support was hydrothermally synthesized from a starting gel having molar ratio: 50SiO_2_:Al_2_O_3_:5Na_2_O:5TPABr:2000H_2_O. The ZSM-5 phase was obtained in stainless-steel Teflon-lined autoclave under static conditions for 120 h at 170 °C. The samples of crystalline mordenite were modified by etching using an aqueous solution of hydrofluoric acid (Sigma-Aldrich) and ammonium fluoride (Sigma-Aldrich). The solution was prepared from 18 mL 0.25 mol/L HF acid, 18 g NH_4_F, and 18 g H_2_O, to which 0.3 g of well-dispersed mordenite crystals were added. The so-obtained mixture was stirred for 20 min at 25 °C and then followed by filtration, washing, and drying at 80 °C [52]. Platinum, palladium, and copper were impregnated on both ZSM-5 materials (initial and acid treated analogues) by incipient wetness technique. The amount of metallic component applied was as follows: Pt = 0.5 wt %, Pd = 0.5 wt %, and Cu = 5 wt %. The metal salts used were: Pt(NH_3_)_4_Cl_2×_H_2_O (Chem-Lab), Pd(NO_3_)_2×_H_2_O (Merck), and Cu(NO_3_)_2×_2.5H_2_O (Chem-Lab).

### 3.2. Characterization

The X-ray diffraction (XRD) powder patterns were recorded on diffractometer PANalytical Empyrean using CuKα radiation. The 2θ scanned range was 4–50 at a step of 0.04° and 2 s acquisition time interval. Nitrogen adsorption-desorption isotherms were obtained applying Quantachrome Instruments NOVA 1200e (USA) at a low temperature of 77 K. Micro-pores contributions were determined by the t-plot method. The surface area was calculated based on the BET equation and the mesopore distribution was evaluated by the BJH method. The high-resolution transmission electron microscopy (HRTEM) images were collected on HR STEM JEOL JEM 2100, equipped with LaB6 electron source at accelerating voltage 200 kV. The specimens were prepared by grinding and dispersing the powders in ethanol by ultrasonic treatment for 6 min. The suspensions were dripped upon standard carbon/Cu grids. The temperature programmed reduction (TPR) measurements were carried out on differential scanning calorimeter (DSC) model DSC-111 (SETARAM) connected to a gas chromatography (GC) with mounted cooling trap (−40 °C) in the gas line prior to the thermal conductivity detector. A hydrogen–argon mixture (10% H_2_), dried over a molecular sieve 5 A (−40 °C), was used at a flowrate of 24 mL min^−1^ and the temperature was linearly raised at a rate of 15 °C min^−1^.

### 3.3. Catalytic Activity Tests

The catalyst samples were tested in reactions of CO and benzene oxidation. The activity of materials in the CO oxidation was tested using an integrated quartz micro-reactor with loading sample amount of 0.5 cm^3^ (fraction 0.63–0.80 mm) and gas chromatograph: Hewlett Packard 5890 Series II. The chromatograph was equipped with a thermal conductivity detector. The inlet gaseous air mixture contained 2 vol.% CO, 10 vol. % O_2_ and N_2_ for balance to 100 vol. %, and GHSVSTP = 40.000 h^−1^. The benzene oxidation tests were performed at atmospheric pressure within a temperature of 100–350 °C and samples amount of 0.5 cm^3^ (fraction 0.42–0.63 mm) placed in a flow fixed bed micro-reactor. The gas mixture passing through the catalysts bed was analyzed by gas chromatograph (Hewlett Packard5890 series II, Wimington, Germany) containing a flame ionization detector with capillary HP Plot Q column. The inlet benzene concentration 42 g m^−3^ in air and space velocity 4000 h^−1^ were used as reaction conditions. The catalysts were pre-activated in flowing pure air for 1 h at 350 °C.

## 4. Conclusions

Synthetic ZSM-5 zeolite is obtained in the system 50SiO_2_:Al_2_O_3_:5Na_2_O:5TPABr:2000H_2_O. It was modified with buffer solution of HF and NH_4_F to obtain a material with secondary mesoporosity. In order to prepare active catalytic materials for oxidation of carbon monoxide and volatile organic compounds (VOCs), platinum, copper, and palladium were loaded by using two types of ZSM-5 zeolite as supports—parent and the same one treated with HF and NH_4_F buffer solution. The catalysts, obtained by use of ZSM-5 zeolites treated with HF and NH_4_F, were more active in the reaction of CO and benzene oxidation compared with the catalyst samples containing untreated zeolite. Both the finer dispersion of metal particles on the hierarchical sample and the presence of secondary mesoporosity played positive roles for increasing the catalytic activity. The creation of additional porosity improved the diffusion of the reagents and minimized the formation of coke. The only exceptions were the copper catalysts in the reaction of CO oxidation, wherein the catalyst sample was more active based on untreated ZSM-5 zeolite. It turns out, that in this case, the key role was played by the oxidative state of copper species loaded on the ZSM-5 zeolites rather than the presence of secondary porosity.

## Figures and Tables

**Table 1:** Textural properties of samples investigated.

Sample	S_BET_^a^ m^2^/g	S_mi_^b^ m^2^/g	V_t_^c^ cm^3^/g	V_mi_^b^ cm^3^/g	V_sec_^d^ cm^3^/g	D_av_^e^ nm
Par	290	242	0.22	0.13	0.09	3.0
Tr	270	212	0.36	0.11	0.25	5.4
Pt-Par	328	277	0.23	0.15	0.08	2.8
Pt-Tr	361	294	0.34	0.15	0.19	3.8
Cu-Par	297	255	0.20	0.14	0.06	2.8
Cu-Tr	319	272	0.24	0.14	0.10	3.1
Pd-Par	318	269	0.22	0.14	0.08	2.8
Pd-Tr	344	276	0.32	0.14	0.18	3.7

^a^ BET surface area. ^b^ Microporous surface area and volume evaluated by the V-t method. ^c^ Total pore volume. ^d^ Secondary meso- and macropores formed by chemical treatment (V_sec_ = V_t_ − V_mi_). ^e^ Average pore size determined by the BJH method.

**Table 2:** Catalytic activity in CO and benzene oxidation.

Sample	90% CO Conv. (T °C)	90% C_6_H_6_ Conv. (T °C)
Pt-Par	211	-
Pt-Tr	189	206
Cu-Par	191	289
Cu-Tr	234	242
Pd-Par	217	258
Pd-Tr	184	249

## Data Availability

Data is contained within the article.

## Sample Availability

Samples of the compounds are available from the authors.
